# An Immuno-Clinic score model for evaluating T cell immunity and predicting early antiviral therapy effectiveness in chronic hepatitis B

**DOI:** 10.18632/aging.202274

**Published:** 2020-12-26

**Authors:** Yurong Gu, Xiaoyan Li, Lin Gu, Yifan Lian, Ke Wang, Youming Chen, Jing Lai, Yongyu Mei, Jing Liu, Zexuan Huang, Min Zhang, Lubiao Chen, Yuehua Huang

**Affiliations:** 1 Department of Infectious Diseases, The Third Affiliated Hospital of Sun Yat-sen University, Guangzhou 510630, China; 2 Guangdong Provincial Key Laboratory of Liver Disease Research, The Third Affiliated Hospital of Sun Yat-sen University, Guangzhou 510630, China

**Keywords:** hepatitis B virus (HBV), Immuno-Clinic score (ICS), T-cell, chronic hepatitis B (CHB), cytokines

## Abstract

We generated an Immuno-Clinic score (ICS) model to evaluate T cell immunity based on the clustering of antiviral cytokines and inhibitory molecules in 229 naïve chronic hepatitis B (CHB) patients. 126 patients receiving antiviral therapy were used to validate the model for predicting antiviral therapy effectiveness. Through receiver-operator characteristic curve analysis, the area under the curve, sensitivity, and specificity of the ICS model were 0.801 (95%CI 0.703-0.900), 0.727, and 0.722, respectively. The cut-off value was 0.442. Re-evaluation of T cell immunity in different phases of CHB showed that patients in the immune tolerant phase had the lowest percentage of ICS-high (15%), while patients in the inactive carrier phase had the highest percentage of ICS-high (92%). Patients in the immune active and gray zone phases had 17% and 56% ICS-high, respectively. Elevation of ICS as early as four weeks after treatment could predict the effectiveness of hepatitis B virus (HBV) DNA loss and normalization of alanine aminotransferase, while eight weeks after treatment could predict HBV surface antigen decline. Thus, this ICS model helps clinicians choose an optimal time for initiating antiviral therapy and predicting its efficacy.

## INTRODUCTION

Despite vaccine and antiviral therapies, hepatitis B virus (HBV) infection remains a global health concern. Over 240 million people worldwide are chronically infected with HBV, which is responsible for 620,000 deaths per year [1, 2]. The disease is slowly progressive in approximately 30% of cases as cirrhosis and 54% of cases as hepatocellular carcinoma [3]. According to current international practice guidelines, the disease phase of chronic hepatitis B (CHB) can be defined by three clinical laboratory parameters that determine the indication for antiviral treatment: alanine aminotransferase (ALT), HBV e antigen (HBeAg), and HBV DNA levels [2]. The factors that determine liver disease severity and their relative importance are not fully defined. Assessment of HBV DNA and ALT might be underrepresented in patients during distinct phases, such as the immune tolerate phase [4–8]. Furthermore, the European Association for the Study of the Liver (EASL) 2017 Clinical Practice Guidelines on the management of hepatitis B virus infection recently published that the “immune tolerant phase” is no longer mentioned in the natural history or new nomenclature for the chronic states [9].

HBV replicates non-cytopathically in hepatocytes and the virus-related diseases are due to chronic, immune-mediated inflammatory events [10]. While the innate branch of immunity is designed for the early stage of infection, T cell immune pathogenesis is the main mechanism for inducing liver injury over a long infection period [11–16]. It is well documented both *in vitro* and *in vivo* that antiviral T cell function is more efficient in patients who can control infection either partially, such as inactive HBV surface antigen (HBsAg) carriers with low levels of virus replication, or completely, such as patients who achieve HBsAg loss either spontaneously or after antiviral therapy [17]. In contrast, a much weaker and barely detectable T cell response is observed during chronic HBV infection. Chronic inflammation alters the access and function of HBV-specific T cells in the liver parenchyma, and also the ability of cytokines to activate antiviral mechanisms [18–22]. Persistent exposure of T cells to HBV antigens is important for maintaining depressed T cell functionality. Loss of cytotoxicity and interleukin-2 (IL-2) production are generally the first to go, followed by tumor necrosis factor-α (TNF-α) and interferon-γ (IFN-γ) production, and ultimately T cell deletion [12]. Besides decreased T cell quantity, negative inhibitory molecules are highly expressed on the functionally exhausted HBV-specific T cells and represent another main cause of dysfunction. These inhibitory molecules include programmed death-1 (PD-1), cytotoxic T-lymphocyte Antigen 4 (CTLA4), lymphocyte activation gene-3 (LAG-3), T cell immunoglobulin domain, and mucin-3 (Tim-3), leukocyte-associated immunoglobulin-like receptor-1 (LAIR-1), and natural killer cell receptor 2B4 (2B4) [23–25].

Although many studies have shown both functional T cells and their inhibitory molecules decide cellular immunity and thus influence clinical and virological features, there is a lack of quantifiable means to identify T cell immunity in clinical practice. We previously developed a practical model to evaluate NK cell immunity in CHB patients [26]. Similarly, in this work we construct a practical Immuno-Clinic score (ICS) model for evaluating T cell status and predicting antiviral therapy efficacy in patients with CHB.

## RESULTS

### Antiviral cytokine and exhausted T cell profiles

**Figure 1:**
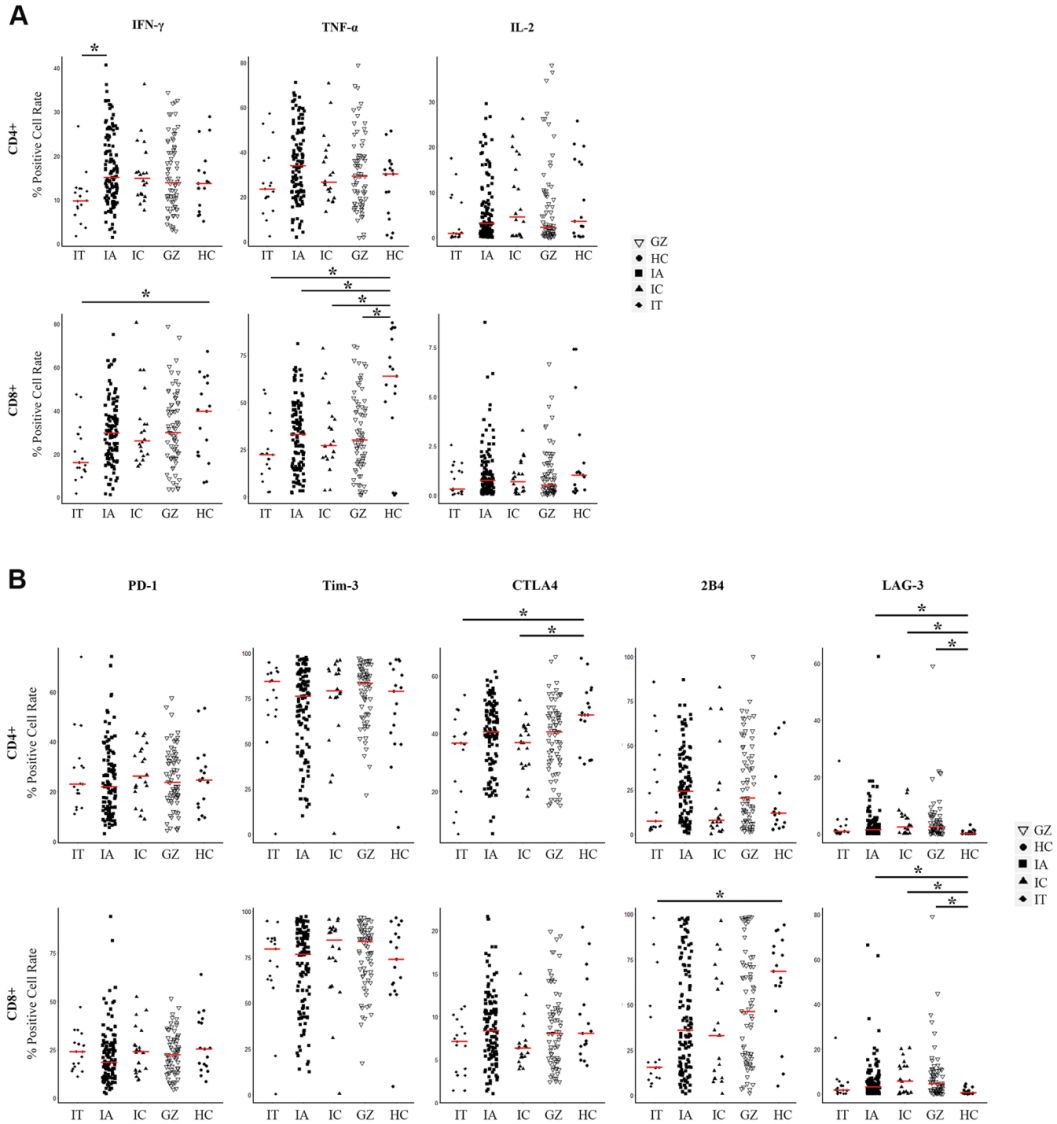
**Expression of three pairs of antiviral cytokines and five pairs of inhibitory molecules by CD4+ and CD8+ T cells from naïve CHB patients.** (**A**) Expression of antiviral cytokines IFN-γ, TNF-α, and IL-2 by CD4+ and CD8+ T cells derived from the indicated patient groups. The levels were compared among patients in the IT, IA, GZ, IC phases and healthy controls. (**B**) Expression of inhibitory molecules PD-1, Tim-3, LAG-3, CTLA4, and 2B4 were measured on CD4+ and CD8+ T cells derived from the indicated patient groups. The levels were compared among patients in the IT, IA, GZ, IC phases and healthy controls. Differences between multiple groups were evaluated by the Wilcoxon rank sum test. Data is presented as the median (indicated by a red line). *P < 0.05.

To display an overall picture of T cell immunity in naïve CHB, we investigated the detailed immune phenotypes of antiviral cytokines and inhibitory molecules in different disease phases. The disease phases of the training cohort were divided into immune tolerant (IT, n=17), immune active (IA, n=120), inactive carriers (IC, n=20), and gray zone (GZ, n=72) phases according to the American Association for the Study of the Liver guidelines, Supplementary Table 1 [2]. The frequencies of antiviral cytokines (IFN-γ, TNF-α, IL-2) and inhibitory molecules (PD-1, LAG-3, Tim-3, CTLA4, and LAIR-1) of T cells are shown in Figure 1. A significant difference was observed in the frequency of IFN-γ produced by CD4+ T cells, with more frequency in the IA patients than in the IT patients (*P* = 0.007). No significant difference in the levels of T cells producing IFN-γ was found between the IA, GZ, and IC groups. Also, TNF-α+ CD4+ and CD8+ T cells were not different in CHB patients for all disease phases. TNF-α+ CD8+ T cells of patients in all disease phases were lower than the healthy controls (*P* = 0.001,0.004, 0.04, and 0.01 in cohorts of IT, IA, IC, and GZ, respectively, Figure 1A). IL-2+ CD4+ and CD8+ T cells were not statistically different between the IT, IA, IC and GZ patients.

To analyze whether CHB had distinct frequencies of exhausted T cells at different disease phases, we measured the inhibitory receptor PD-1, LAG-3, Tim-3, CTLA4, and LAIR-1 expression on CD4+ and CD8+ T cells. Figure 1B shows lower expressions of CTLA4 and LAG-3 in patient groups of IT and IC, and higher expressions of 2B4 in IA, IC and GZ patients than that of the healthy controls. Comparable levels of PD-1 and Tim-3 by T cell subsets were found among the CHB cohort regardless of disease phase and healthy controls.

Taken together, these data suggest that antiviral cytokines combined with co-inhibitory molecules affects the immuno-dominance in HBV infection across CHB disease phases. Therefore, we explored an evaluation model to distinguish comprehensive T cell immunity with both antiviral cytokines and co-inhibitory molecules in CHB.

### Clustering of CHB T cell immunity into immuno-high or immuno-low

**Figure 2:**
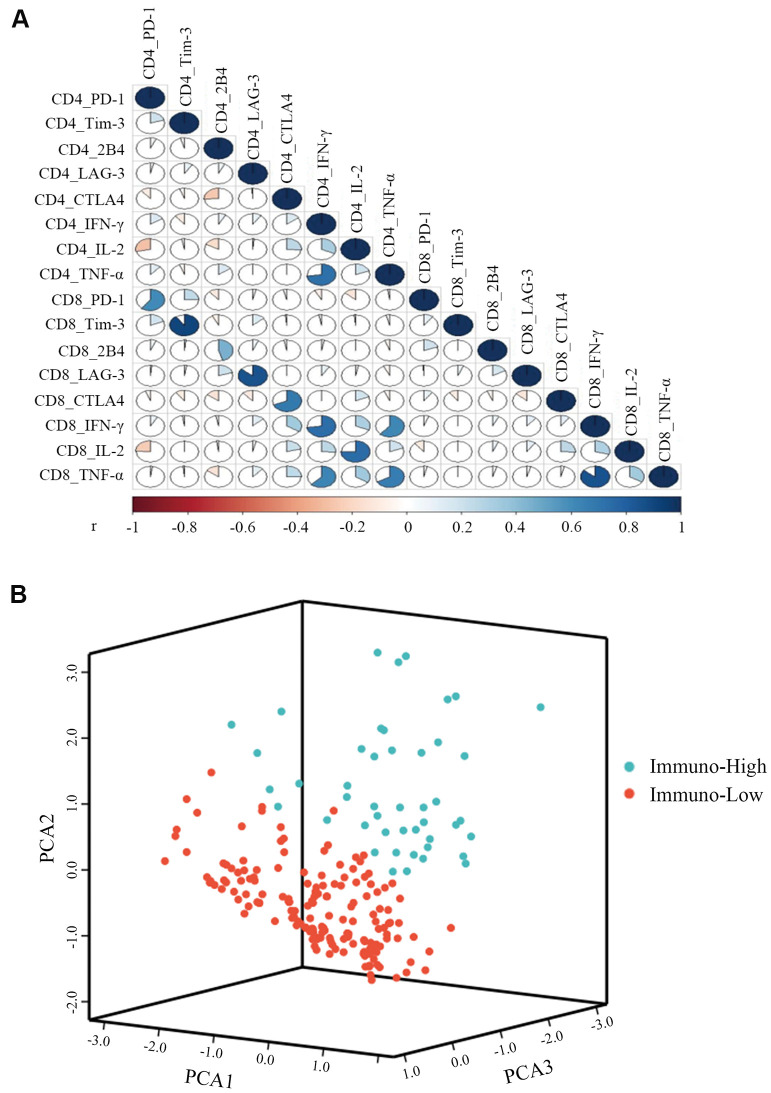
**K-means cluster of CHB patients with three pairs of antiviral cytokines (IFN-γ, TNF-α, and IL-2) and five pairs of inhibitory molecules (PD-1, Tim-3, LAG-3, CTLA4, and 2B4) produced by CD4+ and CD8+ T cells.** (**A**) Correlations among the three pairs of antiviral cytokines and five pairs of inhibitory molecules produced by CD4+ and CD8+ T cells were measured by the Spearman correlation. P<0.05 is colored, and the pseudocolors indicate correlation levels from negative (-1) to positive (1), ranging from a weak (white) to strong (red or blue) association strength. (**B**) Representative image of CHB patient clustering with three pairs of antiviral cytokines and five pairs of inhibitory molecules simultaneously produced by CD4+ and CD8+ T cells. The green balls represent patients of immuno-high (expressing high levels of antiviral cytokines and low levels of inhibitory molecules), and the red balls represent patients of immuno-low (expressing low levels of antiviral cytokines and high levels of inhibitory molecules), respectively. All of the 229 patients were divided into these two groups.

To compare the T cell immunity status of individual CHB patients, we used K-means cluster analysis. Before clustering, the correlation was tested among 16 immune markers for CD4+ and CD8+ T cells, including five pairs of co-inhibitory molecules and three pairs of antiviral cytokines. The data shows that 13 immune markers were independent of each other (r<0.8) and were then used to construct a T cell evaluation model (Figure 2A). Clustering was used to classify 229 patients into two groups of immuno-high or immuno-low. We found significantly greater levels of antiviral cytokines by CD4+ and CD8+ T cells—including IFN-γ, TNF-α, and IL-2—in the immuno-high group than in the immuno-low group (Supplementary Table 2). In contrast, we observed that the expression of exhausted T cell molecules—including PD-1 and 2B4—was higher in the immune-low group. These results suggest that the clustering model could help differentiate between high and low T cell immunity in CHB. The number of patients in the immuno-high group (47, 21%) was less than immuno-low group (182, 79%), indicating that most of the CHB patients’ comprehensive T cell immunity was low (Figure 2B).

### ICS model

**Figure 3:**
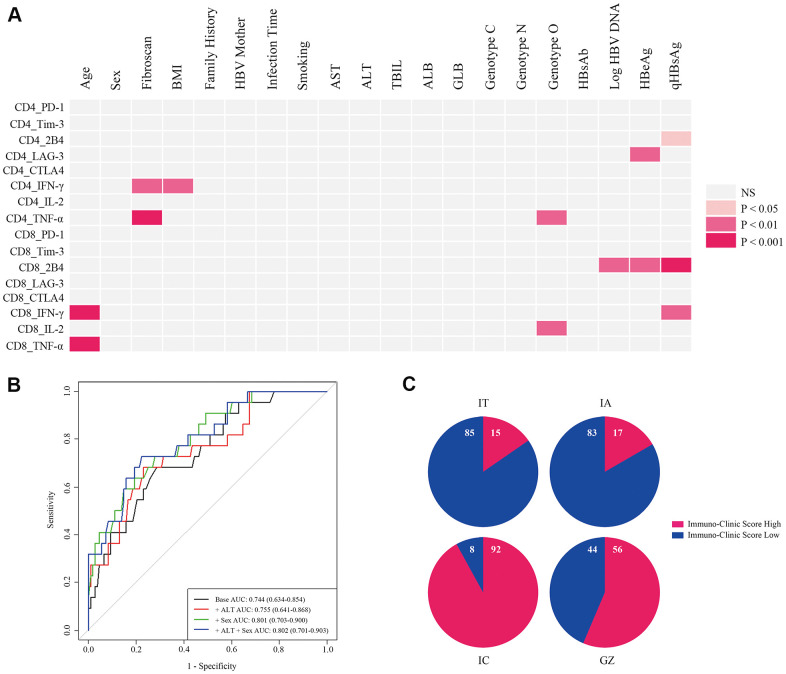
**Development of an ICS model for the evaluation of comprehensive T cell immunity.** (**A**) Correlations of antiviral cytokines (IFN-γ, TNF-α, and IL-2) and inhibitory molecules (PD-1, Tim-3, LAG-3, CTLA4, and 2B4) produced by CD4+ and CD8+ T cells with 18 clinical-virological characteristics were measured. Seven clinical-virological variables (Age, Fibroscan value, BMI, HBV genotype, Log HBV DNA, HBeAg, and qHBsAg) showed significant association with different immune variables. The Spearman correlation or Wilcoxon rank sum test was used to test the correlation. *P* < 0.05 is shown in color, and the red hue depth represents the degree of statistical difference. NS=not significant. (**B**) ROC curve analysis of different ICS models in all CHB patients. Four ROC curves are shown by different clinical-virological variables based on classifications from all CHB patients, and the ROC curve for the selected ICS model is displayed in green (+ Sex). AUC, sensitivity, and specificity of this ROC curve were 0.801 (95% CI 0.703-0.900), 0.727, and 0.722, respectively, and the cut-off value was 0.442. (**C**) Re-evaluation of T cell comprehensive immunity with the ICS model in patients at different CHB phases. Numbers in the blue and red proportion indicate the percentage of ICS-low and ICS-high patients, respectively.

Using clustering plots, we classified patients into immuno-high and immuno-low groups and constructed the ICS model. First, we assessed the clinical characteristics that correlated with immune-markers: Age, body mass index (BMI), HBeAg, Fibroscan value, Log HBV DNA, quantitative hepatitis B surface antigen (qHBsAg), and HBV genotype (Figure 3A). Second, we incorporated the clinical variables into the ICS model. Discrimination was evaluated by analyzing the area under the receiver-operator characteristic (ROC) curve (Figure 3B). We selected the optimal cut-off for the scores based on the clinical variables using the Youden Index. The area under the curve (AUC) of 0.801 (95% CI 0.703-0.900) was generated to discriminate individuals with high T cell immunity from low T cell immunity, with a sensitivity of 72.7% and a specificity of 72.2%. Third, a validated formula was derived for use in clinical practice: ICS= -1.577 + 0.181 Age + 0.088 BMI - 0.373 Fibroscan + 1.363 Sex - 0.250 Log HBV DNA - 0.188 qHBsAg + (1.026 Genotype C + 1.084 Genotype NA - 0.408 Genotype O).

The accuracy of the ICS model was 77.7% by cross-validation (leaving out one validation). The cut-off value for discriminating T cell immuno-high from immuno-low was 0.442. Table 1 shows the scores for each item in the formula**.** Re-evaluation of T cell immunity was performed by the ICS model for patients in different phases of CHB (Figure 3C). In IT patients, 15% were ICS-high and 85% were ICS-low, similar to the percentage of patients in the IA phase where 17% of patients were ICS-high and 83% were ICS-low. In GZ patients, the percentage of ICS-high was 56%, while in IC patients, the percentage of ICS-high was 92%—the highest among the four CHB phases.

**Table 1:** Scores for the selected variables of the ICS model.

**Variable**	**Parameter**	**Score**
Age (y)	< 30	1
	≥ 30 and < 40	2
	≥ 40 and < 50	3
	≥ 50	4
BMI (kg/m^2^)	< 18.5	1
	≥ 18.5 and < 25	2
	≥ 25	3
Sex	Female	1
	Male	2
Fibroscan (Kpa)	< 6	1
	≥ 6 and ≤ 9	2
	> 9	3
Genotype	B	{0,0,0}
	C	{1,0,0}
	NA	{0,1,0}
	O	{0,0,1}
Log HBV DNA (IU/ml)	< 4	1
	≥ 4 and < 7	2
	≥ 7	3
qHBsAg (IU/ml)		
	< 1500	1
	≥ 1500 and < 5000	2
	≥ 5000	3

Immuno-Clinic score = - 1.577 + 0.181 Age + 0.088 BMI - 0.373 Fibroscan + 1.363 Sex - 0.250 Log HBV DNA - 0.188 qHBsAg + (1.026 Genotype C + 1.084 Genotype NA - 0.408 Genotype O).

BMI, body mass index; qHBsAg, quantitative HBV surface antigen; HBV genotype: O-Other included C + D, B + D, B + C, D; NA-not detected.

### Application of the ICS model to predict antiviral therapy efficacy

**Figure 4:**
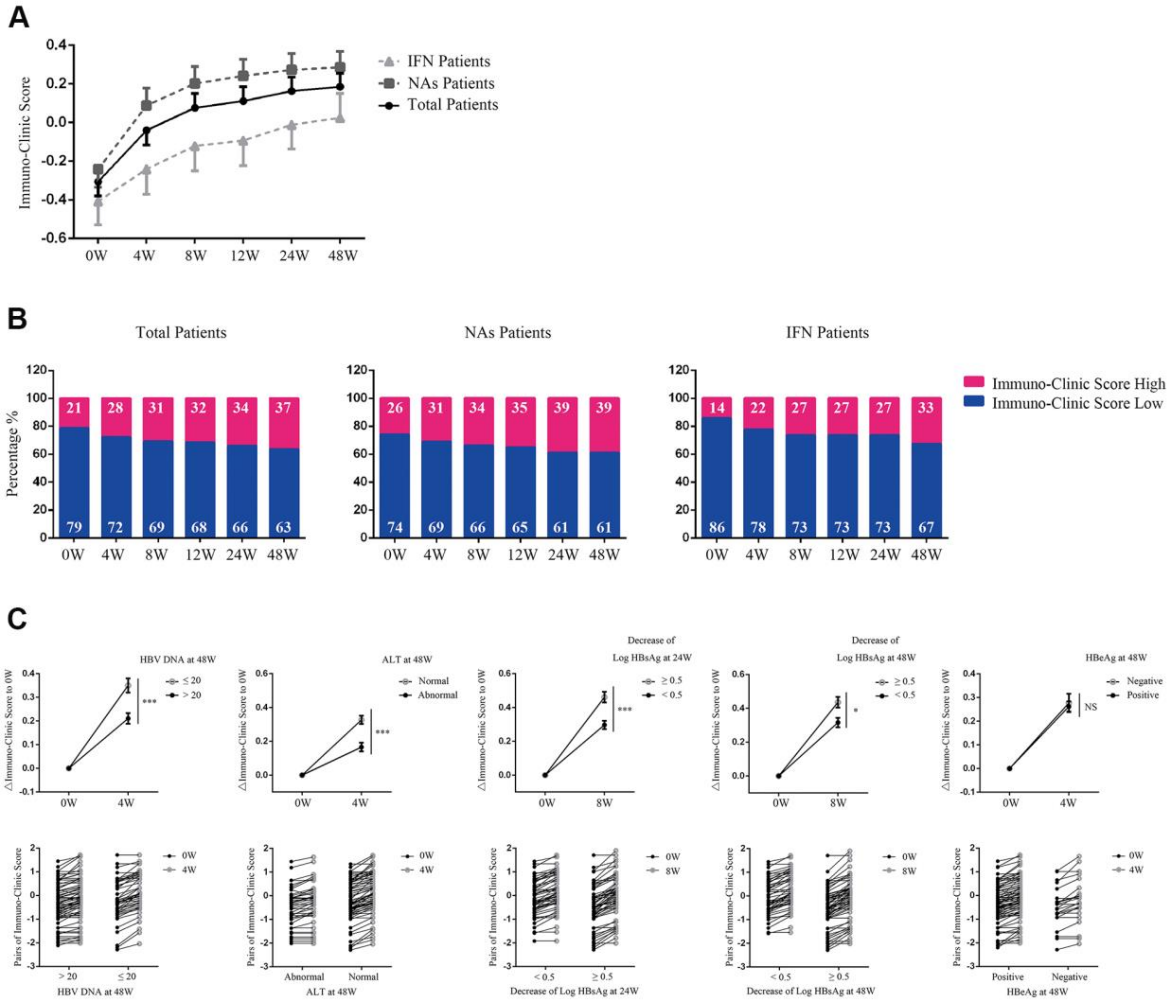
**Application of the ICS model in predicting antiviral therapy efficacy.** (**A**) Evaluation of Immuno-Clinic scores at different time points during antiviral therapy within NAs, PEG-IFN, or total CHB patients. Data are presented as mean ± SEM. (**B**) The percentage of ICS-high or ICS-low patients at different time points during antiviral therapy are shown. Numbers in the blue and red proportion indicated the percentage of ICS-low and ICS-high patients, respectively. (**C)** Comparison of the ICS values between patients with different antiviral efficacy. In the upper panel, ΔICS at weeks 4 or 8 relative to week 0 were calculated. Data are presented as mean ± SEM. ***P < 0.001; *P < 0.05; NS=not significant. In the lower panel, pairs of the original Immuno-Clinic scores for each individual at weeks 4 or 8 and week 0 are shown.

We applied the model to predict antiviral therapy efficacy by longitudinally analyzing the ICS 48 weeks after therapy in 126 CHB patients, including 77 treated with nucleoside analogs [NAs, 70 with Entecavir (ETV), 6 with Telbivudine (LDT), and 1 with Tenofovir (TDF) and 49 with PEG-interferon (PEG-IFN)] (Supplementary Table 3). Figure 4A, 4B shows a progressive increase in the proportion of ICS-high patients during treatment.

Analyses of serologic response revealed that 50 out of 126 patients achieved HBV DNA <20 IU/ml at week 48. Longitudinal data showed a significant increase in ICS as early as week 4, and was found in patients achieving HBV DNA <20 IU/ml at week 48, compared with those who did not (*P*<0.001, Figure 4C). By 48 weeks, 82 out of 126 patients achieved normalization of ALT levels. A significant increase in ICS as early as week 4 was found in patients achieving normalization of ALT levels, compared with those who did not (*P*<0.001, Figure 4C). By weeks 24 and 48, 64 and 68 out of 126 patients achieved a >0.5 log HBsAg IU/ml reduction, respectively. A significant increase in ICS at week 8 was found in patients with a >0.5 log HBsAg IU/ml reduction by week 24 or week 48 compared with those who did not (*P*=0.001, *P*=0.015, respectively, Figure 4C). By week 48, 25 out of 70 patients had HBeAg loss after treatment. However, longitudinal data showed that no significant change of ICS at week 4 was found to correlate with HBeAg loss (Figure 4C).

The main purpose of this study was to construct a practical ICS model to evaluate patients’ comprehensive T cell immunity and use it to predict antiviral efficacy, however, given that both of the HBV DNA and HBsAg levels were included into the ICS model, we compared the prediction efficacy of ICS with HBV DNA and HBsAg levels. The results showed that although the ROCs predicting for HBV DNA and HBsAg decline constructed by HBV DNA and HBsAg themselves had more advantages than ICS, the AUC for ALT normalization at week 48 constructed by Δ0-8w ICS (0.714) was higher than that constructed by Δ0-8w HBV DNA (0.710) and HBsAg levels (0.648). The ROC curve constructed by the Δ0-4w ICS nearly predicted the HBeAg sero-conversion at week 48 (p=0.068, Supplementary Table 4).

These results indicate that a robust increase of ICS as early as week 4 in CHB patients may predict a better outcome of antiviral therapy.

## DISCUSSION

Our data illustrated the different patterns of T cell immunity in 229 CHB patients. By way of clustering, we divided patients into groups of high immunity (immuno-high group) or low immunity (immuno-low group). An ICS model was constructed from the clinical variables. This ICS model can be used to evaluate comprehensive T cell immunity and predict antiviral efficacy, providing liver clinicians an opportunity to identify candidates who would benefit from close supervision or starting antiviral therapy.

We showed that there was consistency between the ICS model evaluation results and CHB disease phases. The patients in the IT phase were thought to be immune tolerant. In the model results, the proportion of ICS-low patients was the highest in the IT phase (85%), indicating that T cell immunity for most IT patients was low. Most of the patients in IC phase (92%) were evaluated as ICS-high, consistent with the traditional concept of IC phase patients. The percentage of ICS-high patients in the IA and GZ phases was in the middle of the four disease phases (17% and 56%, respectively). Re-evaluation results indicated that there were still 15% ICS-high patients in the IT phase, suggesting that although most of the IT patients were ICS-low, there were still ICS-high patients who possessed competent T cell immunity. We found that almost all of the IC phase patients were ICS-high according to the ICS model, which might contribute to the low virus load and normal transaminase seen in IC phase patients.

T cells, including global and HBV-specific cells, play an important role in HBV clearance and are related to liver inflammation from HBV infection [13, 27, 28]. This suggests that evaluating the comprehensive immunity of T cell might benefit disease supervision and antiviral therapy strategies in CHB. Questions regarding treatment initiation and threshold have become more important and controversial [4, 29]. It is widely assumed that waiting to initiate antiviral therapy until the occurrence of clinically active liver disease is an adequate standard of care. However, symptoms are often not apparent until the patient has terminal liver damage. One of the important reasons for not including other HBV-infected individuals beyond the current guidelines is the lack of immune activity or the presence of immunological tolerance. We arbitrarily assumed that ICS-high patients should be given an earlier treatment intervention before irreversible liver damage occurred.

Similar to previous studies that showed HBV DNA and HBsAg as the main factors causing T cell exhaustion [18, 22, 30–33], in the ICS model, HBV DNA and HBsAg also negatively contributed to the model. The ROC results among ICS, HBV DNA and HBsAg levels showed that although HBV DNA and HBsAg had more advantages in predicting their own decline than ICS, the AUC of ICS was higher in predicting ALT normalization, HBeAg sero-conversion, HBV DNA and HBsAg decline than that of HBV DNA or HBsAg alone. Studies have shown that obesity is correlated with T cell immunity. Obese adipose tissue can recruit adaptive immune response-linked CD8+ T cells in mice fed a high fat diet [34]. Inflammatory cells can also accumulate in obesity and result in inflammation. CD4+ and CD8+ T cell infiltration and inflammatory cytokines are also increased in non-alcoholic fatty liver disease (NAFLD) patients [35]. The BMI in the ICS model also positively contributed to the score. Patients who had NAFLD were excluded from our study. In research by Kennedy et al., T cells expressed more PD-1 and secreted less IFN-γ in patients older than 30 [8]. Our study was partly inconsistent with this finding. Age contributed positively to the model, possibly because of younger patients having higher virus loads and levels of HBsAg, both of which depress T cell immunity and negatively contributed to the model.

Sex affects the immune status, especially since the majority of CHB patients are male, and the main endpoint of CHB is also significantly related to sex. There may be two reasons for sex showing no obvious association with immune variables. One was our limited case number (n=229). Chen et al. found that males were associated with elevated baseline HBV DNA levels in a large study (n= 3653) [36]. The other reason is that the immune indicators were analyzed by sex separately, while the model investigated the effect of sex on the global immune status, which could be more informative to show that sex has an impact on immunity. Despite that sex did not show a significant correlation with immune markers, but it still contributed to the global T cell immune cluster, increased the AUC, and made the model more realistic. These results indicate that the ICS model could reflect the advantages of analysis in global T cell immunity.

HBeAg is regarded as a marker for HBV replication and may affect host immunity. HBeAg induces the expansion of monocytic myeloid-derived suppressor cells to dampen T cell function in CHB patients [37] or induce NKG2A natural killer cell dysfunction via regulatory T cell-derived IL-10 in chronic HBV infection [38]. Therefore, the differences of cytokines and exhaustion markers between groups might be explained by the differences in HBeAg levels, as the results showed that HBeAg was correlated with T cell immune indicators. When constructing the ICS model, HBeAg correlated with HBsAg and HBV DNA levels, but did not contribute to the ICS model as much as the latter two. Given that HBeAg is a qualitative variable, it was not adopted into the model. However, HBeAg still affected the ICS and changes in the score could also be explained by a decrease in HBeAg levels during treatment.

With the licensing of PEG-IFN and nucleotide analogues for the treatment of CHB [2, 39], the choice of antiviral therapy has simultaneously become more important and complex. The pros and cons of these drugs as well as patients-specific characteristics should be taken into consideration. Characteristics of ICS-high patients evaluated by the ICS model had low expression of exhausting markers but high secretion of antivirus cytokines, suggesting that PEG-IFN antiviral therapy might be more effective in these patients because their T cell function is preserved, allowing easier induction of antiviral cytokines by interferon. Nucleoside antiviral therapy might be a better choice than interferon for ICS-low patients with a high expression of exhausting markers and low production of antivirus cytokines, because of its weaker antiviral cytokine production potential.

There are some limitations in out study: (1) HBV-specific T cells play a very important role in HBV infection, while the immune response against HBV was not analyzed in this study. We only detected the cytokines and inhibitory molecules of bulk CD4/8 T cells and built an ICS model as a preliminary try based on what we found so far. In future studies, more focus on HBV-specific T cells is needed. (2) Bystander activation of EBV/CMV/FLU affects host immunity and need to be tested in every patient in ideal. It is really a pity that we did not examine these viruses in the study, although none of the patients showed symptoms of EBV/CMV/FLU infection at least at the time of blood samples drawing.

The strengths of this study include the following: (1) Our immune cluster evaluated T cell immunity by combining exhausting markers and antiviral cytokines, which could significantly improve its evaluation ability and integrity. (2) The ICS model was developed from immune clusters of large samples combined with readily available clinical-virological parameters. Our model also avoids the use of expensive and complicated immune tests. (3) ROC analysis indicated that the ICS model evaluation sensitivity was 72.7%, suggesting that most patients with high T cell immunity would be identified by the model. This proportion of patients may benefit from antiviral therapy or being placed under close observation. (4) Longitudinal analysis of antiviral therapy with the ICS model showed that the elevation of Immuno-Clinic scores as early as week 4 could predict the effectiveness of HBV DNA loss, ALT normalization, and HBsAg decline.

In conclusion, we provide a practical ICS model to evaluate T cell immunity in CHB patients. This model can be easily used in clinical practice and serve as a tool to help physicians choose an optimal time for initiating antiviral therapy treatment and predict efficacy. Our immunological data provides a new argument to suggest that patients who preserve a high immune response to viral antigens may be suitable candidates for treatment.

## MATERIALS AND METHODS

### Subjects

Adult patients were recruited from a hepatitis clinic at the Third Affiliated Hospital of Sun Yat-sen University. Written informed consent was obtained from all patients. The study was approved by the Sun Yat-sen University Institute Review Board. Patients were excluded if they received antiviral treatment (interferon or nucleoside analogs [NAs]) within the previous 6 month, had human immunodeficiency virus (HIV), hepatitis C virus, hepatitis D virus co-infection, end-stage liver insufficiency, autoimmune disorders, fatty liver disease, immunosuppressive treatment, cirrhosis, or malignancies.

Two groups of patients were used in the study. The study group (training cohort) included 229 patients with different disease phases for the purpose of developing the above-mentioned T cell profile model. The external group (application cohort) included 126 patients receiving antiviral therapy to confirm this model. The clinical characteristics of these subjects are listed in Table 2 and Supplementary Table 3. In the external group, sequential HBV DNA, HBsAg, HBeAg, and ALT were tested at 4, 8, 12, 24, and 48 weeks after the start of treatment.

**Table 2:** Characteristics of patients and healthy controls included in the study.

**Characteristics**	**CHB (n = 229)**	**HC (n=16)**
Age, years, median (quartile)	29 (25, 34)	27 (25, 44)
Sex n (%)		
Male	161 (70.3)	11
Female	68 (29.7)	5
BMI, median (quartile)	21.5 (19.4, 23.4)	20.8 (19.1, 24.0)
ALT, U/L, median (quartile)	31.0 (23.0, 54.3)	16.0 (13.2, 19.0)
ALB, g/L, median (quartile)	46.1(44.2, 47.9)	45.7 (44.2, 48.0)
TBIL, μmol/L, median (quartile)	13.4 (10.4, 17.0)	9.0 (8.4, 11.8)
Fibroscan, Kpa, median (quartile)	5.2 (4.4, 6.3)	4.5 (4.0, 5.0)
HBeAg status, n (%)		-
Negative	123 (53.7)	
Positive	106 (46.3)	
HBV DNA, Log IU/ml, median (quartile)	5.1 (3.2, 8.2)	-
qHBsAg, Log IU/ml, median (quartile)	3.6 (3.0, 4.5)	-
HBV genotype, n (%)		
B	121 (52.8)	-
C	50 (21.8)	-
O	15 (6.6)	-
NA	43(18.8)	-
Vertical Transmission, n (%)		-
No	177 (77.3)	-
Yes	30 (13.1)	-
Missing	22 (9.6)	-
Smoker, n (%)		
No	203 (88.6)	10 (100)
Yes	26 (11.4)	0 (0)

CHB: chronic hepatitis B. HC: healthy control. ALT, alanine aminotransferase; HBsAg, HBV surface antigen; HBeAg, HBV e antigen. HBV genotype: O-Other included C + D, B + D, B + C, D; NA-not detected.

### Clinical and serologic parameters

HBV e antibody (HBeAb) was tested using commercial kits (Abbott Laboratories, North Chicago, IL). HBV genotype was determined by direct sequencing. Quantitative hepatitis B surface antigen (qHBsAg) was measured by the Elecsys HBsAg II Quant reagent kits (Roche Diagnostics, Indianapolis, IN) according to the manufacturer’s instructions. HBV core antibody (HBcAb) levels were quantified with a chemiluminescence immunoassay (Roche Diagnostics, Indianapolis, IN). Serum HBV DNA levels were measured by Roche COBAS Ampliprep/COBAS Taqman HBV test v2.0 (dynamic range from 20 to 1.7E+08 IU/ml, Roche Molecular Diagnostics, Branchbug, NJ). Fibrosis was defined by liver stiffness measurements (Fibroscan, Echosens, Paris, France).

### Cell surface and intracellular staining

Peripheral blood mononuclear cells (PBMCs) were isolated using Ficoll density gradients. For the phenotypic analysis, PBMCs were stained with FITC-LAG-3, FITC-CD8, PE-2B4, PE-CD8, PE-CTLA4, PE-CF594-CD3, PE-CY7-CD4, BV421-PD-1, V450-CD8 (BD Biosciences, Franklin Lakes, NJ), and APC-Tim-3 (eBioscience, San Diego, CA, USA). For cytokine analysis, PBMCs were stimulated with a Leukocyte Activation Cocktail (eBioscience, San Diego, CA, USA), at 37° C for 4 h prior to intracellular staining using Pharmingen's staining protocol. Anti-human monoclonal antibodies against FITC-IFN-γ, PE-TNF-α (eBioscience), and PB-IL-2 (Biolegend, San Diego, CA, USA) were used. Corresponding isotype-matched controls were purchased from BD Biosciences and eBioscience. Data was acquired on a Gallios instrument (Beckman Coulter, Brea, CA, USA) and analyzed with FlowJo software (Ashland, OR, USA). The gating strategy for cytokines and exhaustion markers is shown in Supplementary Figure 1.

### Statistical analysis

We used a 6-step strategy to develop and validate the ICS model. In step 1, we plotted antiviral cytokines and T cell exhausted molecules in different CHB disease phases by the Wilcoxon rank sum test for continuous variables and χ2 test for categorical variables. In step 2, we tested correlations among 16 immune parameters to identify independent indicators that influenced T cell immunity using the Spearman correlation. In step 3, we used the K-means cluster analysis based on the above selected immune parameters to group parameters with similar patterns of T cell immunity (all the selected immune parameters were scaled in K-means cluster analysis). This method allowed us to partition the data into two groups and develop a T cell evaluation model for classifying individual patients as having high or low T cell immunity. The model was checked through the comparison of immune parameters between the high and low T cell immunity groups. In step 4, the Spearman correlation or Wilcoxon rank sum test was used to test the correlation between the clinical variables and the 16 immune parameters, generating potential clinical variables to be used in the next step. In step 5, discrimination analysis was used to build the ICS models based on the above potential clinical variables and the immunity groups (high or low T cell immunity in step 3), resulting in the Immuno-Clinic scores. The Immuno-Clinic scores and the immunity groups were then used to build the receiver-operator characteristic (ROC) curve. We selected the optimal cut-off for the scores based on the clinical variables using the You den Index. The area under the curve (AUC), sensitivity, and specificity were also computed and compared between the different ICS models. Leave one out validation was also used to confirm the models. In step 6, the selected ICS model was applied to predict the efficacy of antiviral therapy in a longitudinal cohort of CHB patients. ICS model construction and all the other statistical tests were done with R software version 3.2.2 and SAS 9.2. Statistical significance was set at 0.05.

## Supplementary Material

Supplementary Figure 1

Supplementary Tables
