# Supplementary material for: An Immuno-Clinic score model for evaluating T cell immunity and predicting early antiviral therapy effectiveness in chronic hepatitis B
Source: Aging (Albany NY). 2020 Dec 26;12(24):26063–79. doi: 10.18632/aging.202274 (PMC7803537; doi:10.18632/aging.202274)
Supplement: Supplementary Figure 1 [file aging-12-202274-s001.pdf]

SUPPLEMENTARY FIGURE

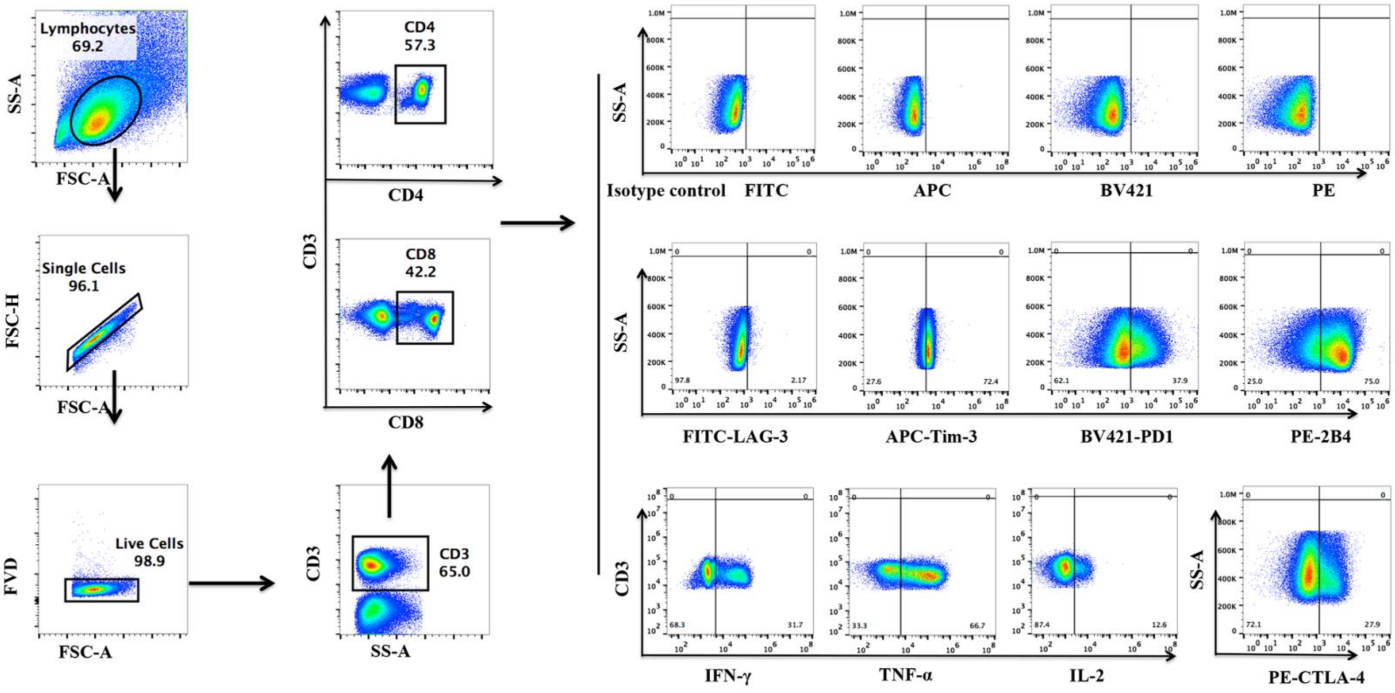

Supplementary Figure 1. The gating strategy for cytokines and exhaustion markers of T cells.
