# Supplementary material for: An Immuno-Clinic score model for evaluating T cell immunity and predicting early antiviral therapy effectiveness in chronic hepatitis B
Source: Aging (Albany NY). 2020 Dec 26;12(24):26063–79. doi: 10.18632/aging.202274 (PMC7803537; doi:10.18632/aging.202274)
Supplement: Supplementary Tables [file aging-12-202274-s002.pdf]

SUPPLEMENTARY TABLES

Supplementary Table 1. Disease phase classification criteria.

Classification	ALT	HBV DNA	HBeAg
Immune tolerance (IT)	Normal	> 1 million IU/mL	Positive
Immune active (IA)	Elevated	>20,000 IU/mL	Positive
		> 2,000 IU/mL	Negative
Inactive CHB (IC)	Normal	Low HBV DNA level	Negative
Grey zone (GZ)	Not classified as IC, IT or IA		

Upper limit of normal (ULN) of ALT: 30 U/L for males and 19 U/L for females.
ALT, alanine aminotransferase; HBeAg, HBV e antigen.

Supplementary Table 2. Expression levels of immunological variables in the immuno-high and immuno-low clusters through four clustering methods.

Variable	immuno-high	immuno-low	P
CD4_PD-1	21.32 ± 0.82	26.13 ± 0.89	***0.03***
CD4_Tim-3	73.73 ± 1.47	72.79 ± 1.45	0.79
CD4_2B4	19.51 ± 1.10	28.45 ± 1.53	***0.00***
CD4_LAG-3	3.96 ± 0.40	3.78 ± 0.48	0.87
CD4_CTLA4	43.82 ± 0.71	36.96 ± 0.79	***0.00***
CD4_IFN-γ	19.77 ± 0.48	14.85 ± 0.53	***0.00***
CD4_TNF-α	37.46 ± 1.05	31.56 ± 1.11	***0.03***
CD4_IL-2	19.45 ± 0.45	3.03 ± 0.21	***0.00***
CD8_PD-1	21.38 ± 0.98	22.62 ± 0.83	0.56
CD8_Tim-3	72.38 ± 1.40	71.26 ± 1.46	0.76
CD8_2B4	43.58 ± 1.89	42.97 ± 2.03	0.90
CD8_LAG-3	7.17 ± 0.76	6.23 ± 0.64	0.56
CD8_CTLA4	9.77 ± 0.25	8.04 ± 0.29	***0.01***
CD8_IFN-γ	38.35 ± 0.97	27.76 ± 1.01	***0.00***
CD8_TNF-α	42.76 ± 1.20	29.04 ± 1.19	***0.00***
CD8_IL-2	2.83 ± 0.11	0.63 ± 0.04	***0.00***

Expression levels indicate the percentage of positive cell. Data are presented as mean ± SEM.
Abbreviation: SEM, standard error of mean.

Supplementary Table 3. Selected clinical-virological characteristics of a longitudinal cohort for the ICS model application.

Characteristics	0W	4W	8W	12W	24W	48W	P value
Treatment: PEG/NAs (ETV, LDT, TDF)	49/77(70, 6,1)						
Age, years, median (quartile)	31 (26, 36)						
< 30, n (%)	51 (40.5)						
≥ 30 and < 40, n (%)	55 (43.7)						
≥ 40 and < 50, n (%)	16 (12.7)						
> 50, n (%)	4 (3.2)						
BMI, median (quartile)	21.1 (19.4, 23.7)						
< 18.5, n (%)	19 (15.1)						
≥ 18.5 and < 25, n (%)	86 (68.3)						
≥ 25, n (%)	21 (16.7)						
Fibroscan, Kpa, median (quartile)	8.7 (6.6, 13.1)						
< 6, n (%)	18 (14.3)						
≥ 6 and <9, n (%)	50 (39.7)						
≥ 9, n (%)	58 (46)						
HBV genotype							
B, n (%)	77 (61.1)						
B + C, n (%)	3 (2.4)						
C, n (%)	46 (36.5)						
HBsAg, IU/ml, median (quartile)	9696 (3472, 25710)	4375 (1821, 15262)	3636 (1551, 8845)	2947 (1332, 8350)	2348(1040, 7728.)	2185 (579, 5830)	**< 0.001**
HBsAb status							0.102
Negative, n (%)	113 (89.7)	103 (81.7)	111 (88.1)	112 (88.9)	106 (84.1)	107 (84.9)	
Positive, n (%)	9 (7.1)	14 (11.1)	13 (10.3)	13 (10.3)	20 (15.9)	19 (15.1)	
Missing, n (%)	4 (3.2)	9 (7.1)	2 (1.6)	1 (0.8)			
HBeAg status							**< 0.001**
Negative, n (%)	14 (11.1)	21 (16.7)	21 (16.7)	22 (17.5)	26 (20.6)	39 (31)	
Positive, n (%)	112 (88.9)	105 (83.3)	105 (83.3)	104 (82.5)	100 (79.4)	87 (69)	
AST, U/L, median (quartile)	81 (57, 129)	58 (39, 91)	41 (31, 60)	36 (28, 55)	29 (23, 41)	26 (21, 38)	**< 0.001**
ALT, U/L, median (quartile)	143 (97, 259)	86 (56, 145)	51 (36, 90)	43 (27, 71)	31 (21, 58)	26 (19, 43)	**< 0.001**
TBIL, µmol/L, median (quartile)	16 (13, 21)	14 (11, 17)	13 (11, 16)	14 (11, 17)	13 (11, 16)	12 (10, 15)	**< 0.001**
HBV DNA, Log IU/ml, median (quartile)	7.96 (6.92, 8.23)	4.61 (3.79, 5.87)	3.6 (2.88, 5)	2.99 (2.33, 4.43)	2.24 (1.61, 3.51)	1.87 (1.3, 3.01)	**< 0.001**
< 4, n (%)	6 (4.8)	37 (29.4)	80 (63.5)	90 (71.4)	103 (81.7)	106 (84.1)	
≥ 4 and < 7, n (%)	29 (23)	72 (57.1)	34 (27)	24 (19)	15 (11.9)	11 (8.7)	
≥ 7, n (%)	91 (72.2)	18 (14.2)	12 (9.5)	12 (9.5)	8 (6.3)	9 (7.1)	
qHBsAg, IU/ml, median (quartile)							**< 0.001**
< 1500, n (%)	10 (7.9)	27 (21.4)	30 (23.8)	36 (28.6)	42 (33.3)	49 (38.9)	
≥ 1500 and < 5000, n (%)	33 (26.2)	38 (30.2)	46 (36.5)	44 (34.9)	44 (34.9)	42 (33.3)	
≥ 5000, n (%)	83 (65.9)	61 (48.4)	50 (39.7)	46 (36.5)	40 (31.7)	35 (27.8)	

ALT, alanine aminotransferase; HBsAg, HBV surface antigen; HBsAb, HBV surface antibody; HBeAg, HBV e antigen.

Supplementary Table 4. Receiver-operating characteristic (ROC) curve comparison among ICS, HBV DNA and HBsAg.

ROC	ALT normalization at week 48				HBV DNA<20 IU/ml at week 48				HBeAg sero-conversion at week 48				HBsAg decline>0.5Log IU/ml At week 24				HBsAg decline>0.5Log IU/ml At week 48			
	**AUC**	**Sen**	**Spe**	***P***	**AUC**	**Sen**	**Spe**	***P***	**AUC**	**Sen**	**Spe**	***P***	**AUC**	**Sen**	**Spe**	***P***	**AUC**	**Sen**	**Spe**	***P***
Δ0-4W HBV DNA	0.702	0.763	0.585	*<0.001*	0.729	0.721	0.723	*<0.001*	0.490	0.821	0.290	*0.861*	0.524	0.275	0.813	*0.610*	0.505	0.617	0.471	*0.909*
Δ0-8W HBV DNA	0.710	0.732	0.642	*<0.001*	0.722	0.656	0.734	*<0.001*	0.453	0.811	0.258	*0.428*	0.597	0.313	0.893	*0.038*	0.576	0.679	0.514	*0.107*
Δ0-4W HBsAg	0.651	0.469	0.784	*0.003*	0.543	0.656	0.527	*0.372*	0.444	0.779	0.300	*0.350*	0.842	0.792	0.840	*<0.001*	0.752	0.722	0.768	*<0.001*
Δ0-8W HBsAg	0.648	0.474	0.818	*0.003*	0.520	0.377	0.769	*0.683*	0.420	0.981	0.067	*0.184*	0.931	0.818	0.933	*<0.001*	0.847	0.797	0.797	*<0.001*
Δ0-4W ICS	0.663	0.742	0.509	*0.001*	0.618	0.361	0.840	*0.013*	0.608	0.745	0.484	*0.068*	0.552	0.800	0.307	*0.276*	0.515	0.063	0.987	*0.757*
Δ0-8W ICS	0.714	0.474	0.906	*<0.001*	0.612	0.492	0.777	*0.019*	0.517	0.991	0.194	*0.779*	0.653	0.686	0.627	*0.001*	0.609	0.646	0.592	*0.026*

Sen, sensitivity; Spe, specificity; ICS, Immune-Clinical Score; ALT, Alanine aminotransferase; HBsAg, HBV surface antigen; HBeAg, HBV e antigen.
